# The economic evaluation of a housing maintenance project to improve the health of Aboriginal housing tenants in NSW: A scoping literature review and protocol for an economic analysis

**DOI:** 10.1016/j.heliyon.2024.e34282

**Published:** 2024-07-09

**Authors:** Simon Deeming, Kerryn Lawrence, Jeffrey C. Standen

**Affiliations:** a Hunter Medical Research Institute, Lot 1, Kookaburra Crescent, New Lambton Heights, NSW, Australia; b School of Medicine and Public Health, University of Newcastle, Callaghan, NSW, 2308, Australia; c Health Protection NSW, Locked Mail Bag 2030, St Leonards, NSW, 1590, Australia; d School of Public Health, Faculty of Medicine and Health, University of Sydney, Camperdown, NSW, 2006, Australia

**Keywords:** Housing for health, Health hardware, Indigenous housing, Aboriginal housing, Economic evaluation, Cost benefit analysis, Cost effectiveness analysis, Policy evaluation, Housing standards, Home health and safety

## Abstract

Considerable evidence exists regarding the role housing plays in the determination of health and well-being outcomes. Despite the scale of health concerns arising from housing considerations, there are very few economic analyses of housing programs that seek to improve health outcomes by addressing the physical infrastructure of the living environment. The NSW *Housing for Health* (HfH) program is an environmental health initiative funded and administered by NSW Health, that addresses health-related hardware in residential accommodation to ensure the home environment supports healthy living practices to ultimately improve health outcomes for residents. This study reviews the economic methods that have been applied to comparable programs and identifies relevant costs and benefits that should be addressed. Founded on the requirement from decision makers, and the insights from the review, the paper outlines a protocol for a cost-benefit analysis that accounts for the disparate health, social, economic and intangible benefits generated from the HfH program and the resources utilised to realise these outcomes.

## 1 Background and objectives

Social determinants, such as access to clean water and sanitation, food, health and social services, income levels, employment, education, transport and housing, have long been acknowledged for their role in health and well-being [[1], [2], [3]]. Significant evidence exists regarding the role housing specifically plays in the determination of health and well-being outcomes [[3], [4], [5], [6], [7]].

Australia's Aboriginal and Torres Strait Islander population has suffered from long-term disadvantage. In response, various Governments have undertaken program and policy initiatives to close the gap between Australia's Aboriginal and Torres Strait Islander and non-Aboriginal population in terms of health, education, economic development, justice, families and young people, culture and heritage, and housing [8,9]. One initiative seeking to reduce this inequality is the *Housing for Health* program.

The broad purpose of this paper is to review the literature to determine the most appropriate method for economic analysis of the New South Wales (NSW) *Housing for Health* program.

### 1.1 The Housing for Health program

The *Housing for Health* (HfH) program is an environmental health initiative that comprises a licensed, multi-stage process of consultation, surveys and housing works. The process aims to assess, repair, or replace health hardware in residential accommodation to enable the residents to conduct healthy living practices (HLPs) in a safe environment. The NSW HfH Program has been predominantly undertaken with Aboriginal community housing providers in remote, regional and urban NSW for over 20 years. The current program is funded by NSW Health and managed by the Aboriginal Environmental Health Unit (AEHU). Previous projects have been funded jointly by NSW Health and the Aboriginal Communities Development Program (ACDP) the Two Ways Together (TWT) initiative, and a variety of other state or Federal programs [10].

The HfH process consists of seven main stages, summarised as.

• Project establishment - Securing funding and working with stakeholders to identify priority communities for project delivery;
• Community consultation and feasibility assessment – Clarification of expectations for the program i.e. includes plumbing, excludes painting; obtain community agreement to implement the HfH project, including a communication plan for unavailable tenants; Completion of a feasibility plan with the relevant social housing provider/s to define the parameters of the project;
• Project preparation – involves informing residents, engaging community workers and arranging consumables and logistics for immediate repair of houses by trades following survey.
• First survey and fix (SF1) – SF1 involves surveying and testing 268 essential safety and health hardware items for every house and yard area using a standardised repeatable and validated survey instrument. The work is completed by teams of local tenants, led by Team Leaders (often from the local Public Health Units). The team members receive prior safety, testing and tool training. During SF1 teams immediately fix any minor repairs not requiring a licensed trade. Works identified as urgent are completed by licensed plumbers and electricians within 1–2 days of the survey.
• Major repairs – Larger or less urgent jobs are prioritised, in accordance with HfH priorities. The Major repairs stage commonly requires a range of service providers, such as a hot water service agent, carpenter, builder, occupational therapist, electrician, plumber, etc.
• Second survey and fix (SF2) - Following the Major repairs stage, SF2 uses the same surveying and repair process as SF1 to audit the works completed, identify any works outstanding or that subsequently arose since SF1 and provide comparative data.
• Reporting and closure – On completion of SF2 works, a HfH final report is submitted to the housing provider containing a list of the work undertaken per house, a forward works list (prioritised in terms of safety and the nine HLPs), project expenditure, and a graph summarising dwelling functionality by defined Healthy Living Indicators at SF1 and SF2 [11].

In the 20 years from 1998 to 2017, 112 HfH projects have been delivered across NSW using this methodology to a total of 3593 houses, including repeat projects in 24 projects (802 houses) [11].

### 1.2 Housing for Health study project

The discrete HfH project that represents the subject investment for the proposed economic evaluation comprises the implementation of the HfH process with a national Tier 1 registered community housing provider managing 127 residential dwellings, in the form of houses and units distributed throughout a rural town in northern NSW. The specific location and name of the study project has been deidentified.

#### 1.2.1 Relevant economic evaluation policy

Funding for the HfH program has been sourced from the NSW Government and Federal Government and directed through NSW Health (AEHU) and delivered in partnership with community housing providers. Consequently, interested decision makers include the respective departments of Health, Social Services and Treasury within both the state and Federal governments. The purpose of an economic evaluation is to inform decision makers regarding the merits of new, recurrent or qualified/refined funding, or to provide grounds for disinvestment. Consequently, the choice of economic method should meet the requirements of the varied decision-makers.

The economic evaluation methods accepted by the NSW government, in common with their national counterparts, include Multi-Criteria Decision Analysis (MCDA), Discrete Choice Experiments (DCE), Cost analysis, Cost-Consequence Analysis (CCA), Cost-Effectiveness Analysis (CEA) including Cost-Utility Analysis (CUA), Cost-Benefit Analysis (CBA), Input-Output Analysis (IO) and Computable General Equilibrium (CGE) modelling [12]. CGE modelling is not insightful for this scale of investment and IO modelling only addresses implications for economic activity. MCDA and DCE are not relevant to ex-ante program evaluation and cost analyses fail to account for the program outcomes. Of the remaining options, CEA/CUA was developed for health economic evaluation. This form of analysis is suitable for economic evaluation of health interventions or programs where the outcomes can be reflected within a single measure, typically a clinical or Health-related Quality of Life outcome. Reference to a single health outcome would not fully reflect the beneficial outcomes from the HfH program. CCA addresses this challenge by reporting multiple outcomes in natural units alongside incremental costs. CCA precludes objective comparison, and consequently assessment against pre-determined thresholds, but is often sufficient for health service decisions. However, the absence of clear decision rules undermines its value for the independent decision makers, such as for treasuries’ common requirement for inter-departmental comparison.

CBA is the preferred method for investment/program/policy evaluation by the Australian Commonwealth and NSW Treasury. CBA is considered a relatively value-free and more comprehensive method with capacity to incorporate overall welfare effects to society, including social, environmental, and economic impacts. CBA is also readily scale-able, to compare different project options and to rank proposals across different policy areas. In addition, NSW Government policy for program evaluation more broadly is shifting from process evaluation to outcomes-based budgeting, where measures of activity levels are only relevant to the extent that they generate measurable benefits [13]. This rationale aligns closely with CBA.

As a consequence of this evaluation policy context, the premise prior to the scoping literature review is that CBA comprises the appropriate form of economic analysis. The results of the literature review sought to inform whether this approach was merited and viable, or whether alternatives provide a more advantageous approach. The literature review also serves to identify considerations that should be addressed and factors that should be incorporated within an economic analysis of such programs.

Guidance from the NSW Treasury recommends that a cost benefit analysis should be completed for capital expenditure exceeding $10 million or more [14]. Investment in the HfH program across NSW totals approximately $2.6 million per year, while the study project totalled approximately $1.0 million over three years. The protocol for the economic evaluation will reflect this discrete study investment but will also demonstrate relevance of an economic evaluation of the statewide HfH program.

The aims for this study were.

• To conduct a scoping literature review: to examine alternative economic evaluation methods applied to programs or interventions designed to improve the health of residents through improvements to physical housing assets associated with human health; to identify key characteristics or factors to be considered within such analyses; and to examine the requirement for relevant evidence given the existing knowledge base. This review is focussed on economic evaluation methods and potential components of these analyses, rather than the identification of specific parameter estimates e.g., effect sizes.
• Founded on the insights from the literature review and prior knowledge of the government policy and program context, prepare a protocol for the economic evaluation of the *Housing for Health* study project.

## 2 Methods

### 2.1 Literature review

To inform the economic analysis, a scoping literature review was conducted in line with PRISMA-ScR guidelines [15]. The appropriate form of literature review was determined using the guidance in Munn, Peters [16]. A scoping review provides for inclusion of policy documentation and relevant non-peer-reviewed publications. Outside of medical and health research, systematic reviews are rarely conducted for most government program evaluations. If the proposed economic analyses demonstrate that the results are critically dependent on key assumptions, such as an improvement in health or reduced hospital admissions, then a systematic review would be appropriate to inform specific parameter assumptions.

*Eligibility criteria* – The search strategy included publications published in English from 1996 to 2019, including Daily, Ahead of Print, In-Process & Other Non-Indexed Citations.

*Information sources* – The bibliographic database/article index *Ovid MEDLINE(R)* was searched. Additional sources were identified from the program managers and through hand searching of references.

*Search strategy –* The search strategy (Appendix A) was conducted on October 2, 2019. Manual follow-up of references and other hand searching was conducted through Calendar Year 2020.

*Selection of sources of evidence* – A more inclusive/heterogeneous approach was used to capture different types of evidence or data sources e.g. peer-reviewed academic papers, government policy documents, third sector reports, etc.

*Protocol for economic analysis* – The protocol was determined with respect to: the need for the economic analysis to provide evidence suitable for the HfH Program Managers, NSW Health and the NSW and Commonwealth Treasuries; and best practice guidelines for the appropriate economic evaluation method.

## 3 Results

### 3.1 Scoping literature review

**Fig. 1:**
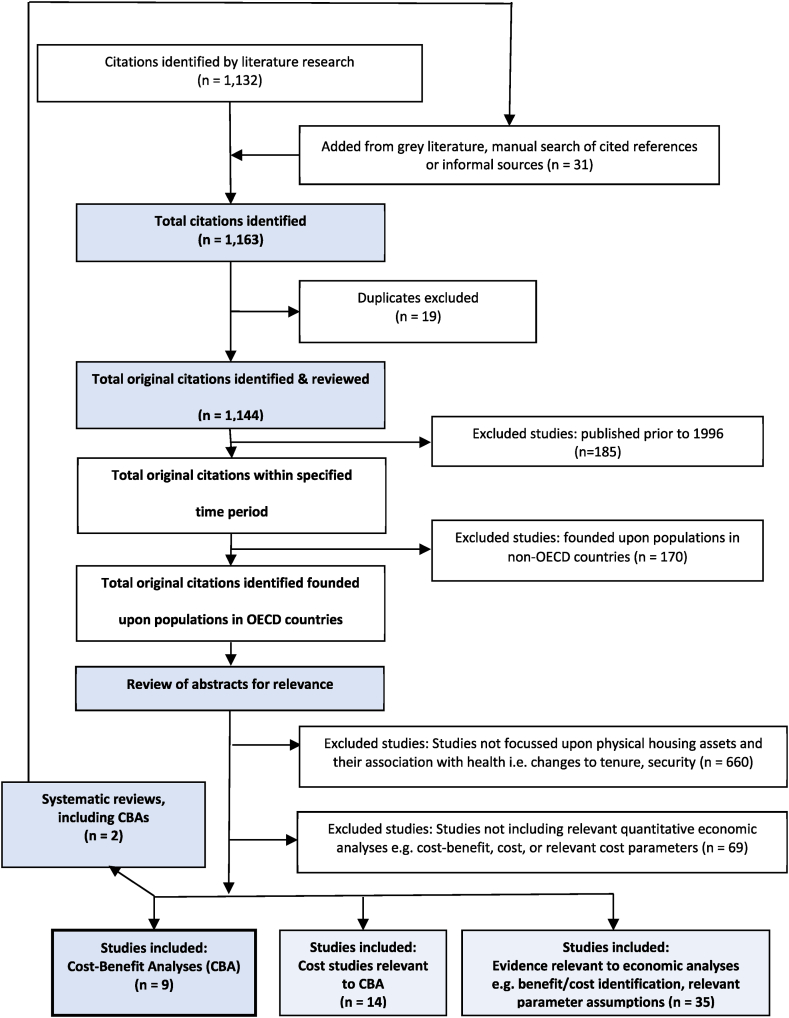
PRISMA-ScR diagram – Literature selection protocol.

The PRISMA diagram for the scoping literature review is provided in Fig. 1. The abstracts were reviewed for relevance to exclude: studies not focussed on physical housing assets and their association with health; and studies not incorporating quantitative economic analyses; or methods directly relevant to the conduct of quantitative economic analyses of *Housing for Health*, or similar programs. The search strategy and manual search captured 1163 potentially relevant citations. The review process identified nine documents containing relevant information regarding cost-benefit analyses of these programs, 14 studies that included cost analyses, and 35 studies that either identified relevant benefit or cost considerations, or information relevant to the method for parameter estimation within an economic analysis.

### 3.2 Physical housing interventions seeking to improve residential health

The economic analyses identified through the literature review which met the intervention criteria included physical modifications to prevent falls, improved ventilation, removal of lead paint, retrofitted insulation to improve domestic temperatures, and smoke alarm installation. These interventions included modifications addressed by the HfH program, but do not include the full range of potential improvements and according benefits. There were no economic evaluations that assessed a program with equivalent focus and breadth.

### 3.3 Economic methods

The literature review identified a range of alternative economic methods applied to relevant programs, including cost-benefit analyses (CBA), cost-utility analyses (CUA), cost-offset analyses, cost-minimisation analyses (CMA) and cost studies. The review identified two relevant systematic reviews. Fenwick, Macdonald [17] conducted a systematic review regarding the economic analysis of the health impacts of housing improvement studies. They identified four CBAs and 25 studies that included costs but had not progressed to an economic analysis. Pega and Wilson [18] conducted a systematic review regarding health economic analyses of housing improvement interventions and insecticide-treated bed nets in the home. They identified 15 CBAs and a further 20 CEA/CUAs. Many of the CBA studies did not meet the specifications of this scoping review because they were either incorrectly termed CBAs or they were conducted in countries outside the search requirement.

Additional studies identified in the systematic reviews were added to the manual search (see Fig. 1 - PRISMA-Scr diagram). In total, eight CBAs were identified for physical housing interventions to address lead poisoning (n = 4), housing insulation (n = 2), risk of falls (n = 1) and smoke detectors (n = 1), and one study addressed the methodological issues of applying CBA to relevant interventions. These evaluations range from poor to excellent quality with respect to formal methods [14,19,20].

Two of the studies were produced by the same research group in New Zealand. The New Zealand studies both assessed the insulation of housing to generate health and other benefits, including an ex-ante CBA of a trial and an ex-post CBA of a government program [21,22]. Their choice of CBA appears driven by OECD and government evaluation policy. The investigators also authored the paper regarding the methodological strengths and limitations of CBA for such interventions. Aside from the policy requirement, CBA was acknowledged for its capacity to counteract narrow government thinking and special interest lobbying, for its applicability irrespective of scale, for its inclusion of disparate costs and benefits that may otherwise be ignored, and as a framework to articulate value [23]. Their application of CBA identified a number of specific challenges including the theoretical assumption that utility can be transferred from winners to losers, the risk of bias towards considerations for which strong evidence is available, particularly for impacts with low population rates e.g. hospitalisation, the valuation of health, well-being and mortality, and the treatment of inter-generational and societal equity considerations [23].

A CBA was also conducted by Nevin, Jacobs [24] to evaluate an intervention to remove indoor lead paint. The implications of lead paint poisoning are broad, particularly for children, and consequently, their analysis (and the update by Dixon, Jacobs [25]) accounts for potential health service, social and economic benefits.

Gould [26] conducted a model-based CBA of indoor lead paint removal. As per Nevin, Jacobs [24], a CBA framework enabled the analysis to account for a wider range of healthcare, economic and social impacts, although they did not incorporate a value for health outcomes. The Pichery, Bellanger [27]'s partial CBA of lead hazard control valued similar societal benefits. Their analysis sought to value imposts on quality of life via a judicially-determined compensation methodology.

Ling, Henderson [28] accounted for costs and benefits of a home modification program to prevent falls. The formal quality of the CBA was low. Their study included reductions in aged care admissions, which represents a potential benefit from the HfH program, due to the inclusion of this factor within the survey/modifications. Finally, Liu, Mack [29] evaluated a smoke alarm intervention using a CBA. Their evaluation adopted a CEA framework, but sought to monetise health outcomes, via quality of life loss, as well as tangible cost savings, such as health care savings, economic productivity gains and property savings. As per Pichery et al., their valuation of health-related quality of life, utilised evidence from jury verdicts and settlements for fire injuries. A Markov state transition model was used to estimate the home fire incidence rates, mortality rate per fire and accordingly the mortality risk reduction from smoke alarms. A value was placed on mortality using estimates for Life Years Saved (LYS) and the Value of a Statistical Life Year (VSLY).

Preval, Chapman [30] also conducted a CBA that met the search criteria, but the intervention, provision of residential heaters, is not a component of the HfH program. Laing and Baker [31] were categorised as a CBA in Fenwick, Macdonald [17], but no evidence of the outcome methods, measures or values could be sourced.

The primary conclusions arising from the Fenwick systematic review were that public health initiatives regarding housing interventions should, but rarely, include economic evaluations. It also identified the need to collect data over an extended period to capture longer term impacts, the need to plan for economic data collection from commencement of the program, and the need to adopt a broad perspective to capture the disparate costs and benefits that may arise from housing interventions [32].

Alternative economic evaluation methods identified through the scoping review included: cost studies that seek to estimate the economic costs arising from poor housing e.g. Chenoweth, Estes [33]; cost-offset studies, which estimate the costs of public housing investment net of potential fiscal savings e.g. Davidson, Nicol [34]; cost-effectiveness analyses (CEA), which estimate an incremental cost per change in a single health outcome arising from an intervention e.g. Coskeran, Denman [35]; cost-utility analysis (CUA), a CEA for which the Health related Quality of Life (HrQoL) outcome measure has been converted to a Quality Adjusted Life Year (QALY) e.g. Frick, Kung [36], and cost-consequence analyses (CCA) e.g. Bray, Burns [37], which report multiple outcomes in natural units, alongside the costs for an intervention. The results of most of these analyses were presented as evidence to inform program funding. However, the selected methods were not ostensibly determined by the funders’ preferred methods.

### 3.4 Relevant economic cost considerations

Table 1 lists the cost categories identified within all the economic studies found through the broader literature search. The capital costs of physical interventions were included in all the studies. Program and project management costs were included in all studies, albeit often incorporated within aggregate program costs. Privately contracted specialist components were detailed for few studies [38]. Grimes, Denne [22] included the producer surplus estimate arising from the additional public expenditure, the value of which they deducted to derive net program costs. This economic cost consideration is only relevant for CBAs.

**Table 1:** Potential incremental costs associated with health/physical housing initiatives and corresponding source for methods/parameter estimation.

Attribute	Attribute source	Relevant considerations
Program management (incl admin)	All (explicit only in some)	Majority of studies utilised primary data source for subject program cost
Project management	All (explicit only in some)	Majority of studies utilised primary data source for subject program cost
Capital costs of physical infrastructure	All	Majority of studies utilised primary data source for subject program cost
Contracted specialist services	Explicit only in Haddix, Mallonee [38]	Primary data source
Producer surplus benefits arising from public expenditure	Grimes, Denne [22]	Deducted from program costs to derive a net cost in Grimes, Denne [22]
Volunteer time (residents)	Liu, Mack [29], Haddix, Mallonee [38]	Lost leisure time; potentially valued at $0 for excess leisure time
Paid employment (residents)	Haddix, Mallonee [38]	
Deadweight loss from taxation	Grimes, Denne [22]	Relevant to all programs; included in few
Administration of taxation/public insurance	Grimes, Denne [22], Liu, Mack [29]	Relevant to all programs; included in few
Educational material	Haddix, Mallonee [38], Phillips, Humphreys [39]	Example of implementation costs

Two studies accounted for costs attributable to roles within the intervention conducted by residents, either paid or volunteered [29,38]. These costs were not relevant to every program intervention. However, the formal scope/study boundary for many of these studies was poorly defined and consequently, the grounds for exclusion was often unclear. Implementation costs, such as the provision of educational materials, were only identified in two studies [38,39].

Economic cost considerations such as the deadweight loss/excess burden of taxation and the cost of administering taxation/public insurance were included in two of the 12 CBAs [22,29]. These economic costs are only relevant to CBAs.

### 3.5 Relevant benefits

The literature review identified 19 potential benefit categories across all the identified studies (Table 2). The respective studies demonstrate methods potentially relevant to estimate the value of equivalent benefits from the HfH program.

**Table 2:** Potential incremental benefits associated with health/physical housing initiatives and corresponding source for methods/parameter estimation.

Attribute	Attribute source^a^	Relevant considerations
Healthcare – Primary care (General practitioners) services	Chapman, Howden-Chapman [21], Grimes, Denne [22], Nevin, Jacobs [24], Gould [26], Pichery, Bellanger [27], Liu, Mack [29], Preval, Chapman [30], Chenoweth, Estes [33], Bray, Burns [37], Barton, Basham [41], Salkeld, Cumming [42], Brown [50]	Barton, Basham included health service saving on Cost side; Grimes, Denne [22] modelled estimates based on other studies
Healthcare – Reduced hospitalisations/Emergency Department attendance	Chapman, Howden-Chapman [21], Grimes, Denne [22], Nevin, Jacobs [24], Gould [26], Pichery, Bellanger [27], Liu, Mack [29], Chenoweth, Estes [33], Bray, Burns [37], Haddix, Mallonee [38], Phillips, Humphreys [39], Barton, Basham [41], Salkeld, Cumming [42], Ginnelly, Sculpher [49], Han, Ungar [51], Nicol, Roys [52]	Barton, Basham [41] included health service saving on Cost side
Healthcare – Medication (explicit from health services)	Liu, Mack [29], Preval, Chapman [30], Barton, Basham [41], Salkeld, Cumming [42]	Medication costs not separated but incorporated into healthcare cost estimates for many projects
Health & Well-being – Health-related Quality of Life improvements/Morbidity averted	Chapman, Howden-Chapman [21], Pichery, Bellanger [27], Chenoweth, Estes [33], Frick, Kung [36], Bray, Burns [37], Church, Goodall [40], Barton, Basham [41], Salkeld, Cumming [42], Lawson, Kearns [43], Keall, Ormandy [44], Franchimon [45], Jutkowitz, Gitlin [46], Coskeran, Denman [53]	EQ-5D: Coskeran, Denman [35], Bray, Burns [37], Church, Goodall [40] Short Warwick-Edinburgh Well-being Scale (SWEMWBS): Bray, Burns [37] SF36: Barton, Basham [41], Salkeld, Cumming [42], Lawson, Kearns [43] GHQ12: Barton, Basham [41] Literature-derived Quality Adjusted Life Years (QALYs): Liu, Mack [29], Frick, Kung [36], Phillips, Humphreys [54] Financial compensation method: Pichery, Bellanger [27] Disability Adjusted Life Years (DALYs): Chenoweth, Estes [33], Keall, Ormandy [44], Franchimon [45]
Health – Reduced mortality/Life Years Saved	Chapman, Howden-Chapman [21], Grimes, Denne [22], Liu, Mack [29], Jutkowitz, Gitlin [46]	Life Years Saved (LYS) (no. of days to death; difference in area under Kaplan-Meier survival curve estimated LYS); Double-counting must be avoided if long-term productivity losses/gains incorporated
Social – Reduced demand on emergency services, coroner services, funeral costs (e.g. fire)	Liu, Mack [29], Haddix, Mallonee [38], Ginnelly, Sculpher [49]	
Social – Informal care (e.g. fall related; childcare)	Preval, Chapman [30], Salkeld, Cumming [42]	Double counting must be avoided with any well-being benefits
Social – Reduction in long term care (e.g. fall related)	Kochera [48]	Predominantly falls related, with other long term benefits less directly attributable to the intervention
Social – Reduced aged care admissions	Ling, Henderson [28]	Predominantly falls related, with other long term benefits less directly attributable to the intervention
Social – Reduced crime	Nevin, Jacobs [24], Gould [26], Pichery, Bellanger [27]	Related to education outcomes
Social – Education: Days off school/value of lost education/economic productivity loss to children's parents	Chapman, Howden-Chapman [21], Grimes, Denne [22], Preval, Chapman [30], Barton, Basham [41], Brown [50]	Predominantly modelled analysis founded on parameter estimates from other studies
Social – Education: Reduction in Special education costs	Gould [26], Pichery, Bellanger [27], Brown [50]	
Economic – Utilities savings (electricity, water)	Chapman, Howden-Chapman [21], Nevin, Jacobs [24]	
Economic – Changed asset management/maintenance costs	Franchimon [45]	Inclusion in costs or benefits dependant on program intervention
Economic – Patient & carer transportation and lost time	Nevin, Jacobs [24], Ginnelly, Sculpher [49]	Double counting must be avoided with any well-being benefits
Economic – Additional employment (direct investment stimulus)	Grimes, Denne [22]	Need to ensure incremental to counterfactual i.e. alternative investment in other economic activity that may generated employment; Probably list as intangible for study project
Economic – Productivity loss/gain (short term due to health interruption to employment, employer borne; value of lost housework)	Liu, Mack [29], Haddix, Mallonee [38]	
Economic – Productivity loss/gain (lifetime earnings, excl education)	Nevin, Jacobs [24], Gould [26], Pichery, Bellanger [27], Haddix, Mallonee [38]	Double-counting must be avoided if mortality valued using LYS/VSLY method
Economic - Additional tax revenue	Gould [26]	Double counting must be avoided if producer surplus considerations included
Environmental – CO_2_e savings	Chapman, Howden-Chapman [21]	Within a societal perspective, relevant to renovations that affect heating and/or air-conditioning

a Notes: Sources represent examples of the inclusion of the respective attribute within the broader literature review results, including studies adopting non-CBA methods.

#### 3.5.1 Healthcare service benefits

Healthcare, particularly net health service savings, dominated the potential benefits within most studies, demonstrating the importance of this component within economic analyses of comparable housing interventions (full reference list in Table 2). Methods to estimate the impact upon primary healthcare services e.g. general practitioners, changes in hospital admissions and Emergency Department attendances, and medication use (often incorporated within health service costs) varied from primary studies to model-based analyses using secondary data.

#### 3.5.2 Health benefits

CEA/CUA frameworks provide a stronger theoretical bases for the inclusion of health outcomes within economic analyses. Consequently, the majority of studies that estimated program benefits for HrQoL, well-being, pain and suffering, or morbidity effects were conducted using these methods. For the studies identified through the review, a number of different instruments were used to measure these health outcomes, including EuroQoL EQ-5D [35,37,40], 36-Item Short Form Survey (SF-36) [[41], [42], [43]], General Health Questionnaire-12 (GHQ-12) [41], and the Short Warwick-Edinburgh Well-being Scale (SWEMWBS) [37]. Some studies used these instruments to estimate gains in QALYs, while others applied estimated QALY gains evidenced within the published literature.

Three studies utilised Disability Adjusted Life Years (DALYs) to estimate morbidity effects [33,44,45]. These analyses typically utilise environmental attributable fractions to estimate both the direct and indirect health service costs, and the DALYs, associated with environmental conditions.

Some studies directly accounted for a reduction in mortality attributed to the respective interventions [21,22,29,46]. Evidence of reductions in mortality attributable to the intervention were combined with assumptions regarding respective survival curves for the control and intervention to estimate the number of Life Years Saved. One study, Barton, Basham [41], measured a range of clinical respiratory and musculoskeletal health outcomes that were reported alongside HrQoL outcomes in a CCA format.

#### 3.5.3 Social benefits

The social benefits included within the identified studies fall into four categories: short term benefits that arise from improved health, including reduced childcare demands, days off school [21,22,30,41,47]; positive long term benefits that arise from improved health, such as reduced special education needs, improved educational outcomes, reduced crime and the economic productivity gains that arise from improved life trajectories [21,22,26,27,29,46,47]; benefits from reduced falls, including informal and formal aged care demands, reduced aged care admissions [28,48]; and benefits from a lower probability of fire, such as the reduced resources required for emergency services, coroner services and funerals [29,38,49].

#### 3.5.4 Economic benefits

The economic benefits within the identified studies included direct impacts, such as utilities savings (electricity, water) [21], reduced asset management/maintenance costs [45], and the additional employment arising from the program investment [22]. The indirect economic benefits include the productivity gains arising from reduced workplace absence [29], net gains to domestic work [38], and the societal gains reflected in improved lifetime earnings resulting from improved health outcomes [24,26,27,38].

Two studies also accounted for reductions in out-of-pocket patient & carer transport costs and the time lost attending health services [24,49].

#### 3.5.5 Other benefits

The environmental benefit from improved housing insulation comprised a significant ancillary benefit for interventions seeking to improve health via improvement in indoor temperatures [21].

### 3.6 Benefit valuation

Many of the potential benefits identified in the literature were not valued, but reported as changes in natural units, consistent with the CUA, CEA or CCA framework. Exceptions include the cost-offset studies, which valued projected net health service savings to offset against investment costs [34]. Some of the CUA studies introduce an assumed value per QALY/DALY to inform an investment decision rule, but this approach only informs investment decisions against comparators evaluated using the same theoretical framework.

Within the CBA studies, five excluded the potential value of health benefits due to either a lack of data regarding potential changes in quality of life, well-being or morbidity, or the methodological challenges associated with their valuation and inclusion within this theoretical framework. Of the three studies that quantified values for health benefits, Pichery, Bellanger [27] utilised a legal compensation method to value potential gains in morbidity, Grimes, Denne [22] valued projected gains in mortality using the Value of a Statistic Life applied to LYS, while Liu, Mack [29] incorporated values for both mortality and morbidity using both methods.

### 3.7 Equity

The results demonstrate that most of the identified studies were positioned within an equity policy context that seeks to improve outcomes for marginalised and low socio-economic status community cohorts. However, none of the relevant economic evaluations specifically accounted for equity within the quantitative component of the analysis.

### 3.8 Protocol for the economic evaluation of the HfH study project investment

Founded on discussions with the project delivery team within the Public Health Unit of the relevant Local Health District, the Program's managers (the Aboriginal Environmental Health Unit within NSW Health), the respective Centres for Evidence and Evaluation (CEE) within NSW Health and the NSW Treasury, and the insights from the literature review, the following section outlines a protocol for the economic evaluation of the HfH study project.

*Title:* Prioritising housing maintenance to improve health in Aboriginal communities in NSW: an ex-post cost benefit analysis of a *Housing for Health* Project

*Purpose –* The primary stakeholders for the economic analysis are the Program Managers within the AEHU, the community housing provider, NSW Health and the NSW Treasury. The purpose is to conduct an economic evaluation of the project/program to inform decision makers regarding the relative merits of on-going funding.

*Target population and setting –* Given that the costs and benefits from the HfH study project extend beyond the resident population, the target population comprises the broader community within the Local Health District catchment.

*Choice of economic framework –* The proposed economic analysis will comprise an ex-post cost benefit analysis (CBA). The choice of economic framework is informed by the format required by the decision makers to optimise the outcomes from public investment into public health [12].

*Study perspective* – the economic analysis will adopt a societal perspective consistent with a social welfare cost-benefit framework.

*Program/project comparator* – Application of the seven-stage HfH program to the study project for the period from July 2018 to June 2020. The study project addressed 127 dwellings providing housing for 302 residents. The project identified and repaired 2467 items.

*Baseline comparator* – An ex-post CBA requires a counterfactual reflecting the baseline against which the incremental changes in societal outcomes and costs can be referenced. The baseline will reflect a modelled scenario in the absence of the implemented HfH program. Evidence for the counterfactual will be derived from the available pre and post intervention data for the affected residents and from the available literature.

*Study design/model* – The extent to which the effects can be attributed to the program will be primarily examined via a marginal analysis using a pre-post design.

### 3.9 Benefit estimation

Potential economic benefits identified through the literature review will each be considered for inclusion founded on: the relative size of the potential benefits; the strength of evidence, primary or secondary, attributing the outcome to the program intervention; and the potential magnitude of the benefits relative to the research cost of deriving the estimate. Impacts on healthcare service use will be explicitly incorporated. Where evidence exists, mortality gains will be included, but any potential health, well-being and morbidity effects will be addressed within the sensitivity analyses.

To retain consistency with costs, productivity gains will be valued using the human capital method. To retain the confidence of primary stakeholders in the results, a conservative approach will be adopted to the inclusion and valuation of any benefits, and transparency will be provided to the evidence base.

Components not monetised will be retained as intangible outcomes alongside the reported results. For example, the capacity for Aboriginal elders to remain in community carries significant cultural and social value. It is critical that such intangible values are acknowledged with equivalent merit to the elements more readily monetised.

### 3.10 Resource and cost estimation

The resource components required to generate the benefits include: program management, project management and administration, contract management, coordination, training, work audits, reporting and closure; survey conduct and data administration; team repairs; licensed trade repairs; tasks conducted by residents, including both paid and volunteered time; and investment in the hardware. The opportunity cost of in-kind support from AEHU, the LHD, the community housing provider, the local Aboriginal medical service and the local government council will also be incorporated. Any additional resources will be identified through the CBA process. The costs will be derived from shadow prices for labour and equipment, and market prices for materials and outsourced contracts e.g. licensed repairs. Estimates for the resource utilisation will be derived from records of direct expenditure, administration records or manager estimates for opportunity costs.

The resources required to derive estimates for some components, such as the beneficial offset from additional producer surplus, prohibit inclusion within the project evaluation, but could be incorporated within a wider evaluation of the whole HfH program.

*Time horizon* - The time horizon for the economic analysis will comprise ten years from approval of the memorandum of understanding between AEHU and the community housing provider in July 2018. This period will account for the resources required to prepare and consult with the community, as well as the period of investment into housing maintenance through to the reporting and closure stage of the program. The time horizon extends beyond this period to account for the generation of downstream benefits and any costs that may be necessary for the realisation of on-going benefits.

*Discount rate -* NSW Treasury recommend the application of the social opportunity cost of capital, which is currently estimated at 7 %. NSW Treasury are also keen to maintain consistency across past and present economic evaluations, which utilised a 7 % discount rate [12]. There is concern that the adoption of this discount rate discriminates against projects with longer term benefits, particularly where greater uncertainty exists regarding higher long-term benefits and/or costs. Consequently, this evaluation will conduct sensitivity analyses with 3 %, 5 %, 7 % and 10 % discount rates.

*Currency and price date* – Specification of the currency and price date represents good practice but will also contribute to NSW Treasury's goal to build the comparable evidence base over time.

*Analysis and reporting* – The analysis will report a Benefit Cost Ratio and Net Present Value of the HfH study project. Sensitivity analyses will be conducted to identify the key assumptions and their impact on the decision rule. In line with the purpose to inform key stakeholders, the economic evaluation will be reported according to the NSW Government guidelines for CBA.

*Accounting for equity –* Equity is an important consideration for this HfH program, given the highly disadvantaged profile of the resident population. Equity considerations will not be incorporated directly into the quantitative analysis, due to the absence of a commonly accepted method. Rather, the results of the economic analysis will be positioned within wider distributional policies to provide for comparison to alternative programs or investments seeking to realise similar equity objectives.

## 4 Discussion

To optimise the public benefits generated through investment in publicly-funded programs, policy makers and public treasuries are increasingly focussed on a program's outcomes rather than demonstration of activities [13]. This policy is commonly accompanied by a requirement to conduct appropriate economic analyses that provide transparency to the value generated from the investment to enable comparison and optimisation across public budgets [12,55]. The requirement for such economic analyses is likely to increase as fiscal budgets are constrained to recover from the public debts incurred as a result of the Global Financial Crisis and COVID-19 recession.

With respect to the study aims, this review supports the requirement for an economic analysis of the HfH study project. Despite the scale of health concerns arising from physical housing considerations, and despite the scale of public investment directed towards this issue, the literature review identified very few economic analyses of housing programs that seek to improve the physical infrastructure for health reasons.

The review identified that a range of economic evaluation methods have been applied to comparable program investments. With respect to the program's stakeholders, treasuries prefer CBA to inform resource allocation between alternative public investments [12,19,20,23]. Where health outcomes can be reflected in a single measure, health departments more commonly utilise CEA/CUA to inform on resource decisions for alternative health investments [36,56].

The scoping review of the literature identified nineteen potential benefits from the HfH study project. Of the alternative economic evaluation methods utilised only CBA or CCA provide a consistent framework to include these disparate outcomes. While accepted methods exist to incorporate mortality gains within CBA, challenges exist with the valuation of health, well-being, or morbidity gains. The compensation approach utilised by Pichery, Bellanger [27] and Liu, Mack [29] is not supported within the government guidelines relevant to the program [57]. CUA/CEA was designed to evaluate health outcomes but excludes most other benefits from the quantitative analysis. The review identified net health services gains as a large potential benefit, but this is readily incorporated within most methods. Given these considerations, the protocol retains a CBA framework providing the capacity to scale the analysis to an evaluation of the wider HfH program.

The primary challenge arising from the application of CBA lies with the exclusion of potential health gains from the evaluation of a health program. Some government guidelines outline accepted methods to value health [57], although these are often inconsistent with the social welfare theory foundations of CBA [58]. Consequently, to account for any evidence demonstrating improvements in residents’ health, well-being or morbidity, relevant values will be included within the sensitivity analysis. This approach can inform the decision rule, as per CUA, without undermining theoretical consistency within the central analysis.

A second challenge arises with the number of potential benefits that represent improvements in low probability but high value events, such as hospital admissions, mortality from fire, falls and aged care admissions. Primary data is required to demonstrate a program effect on lower probability events, preferably from commencement of the program, and preferably over a long period [32]. Such evidence is unlikely for small investments like the HfH project. To capture these benefits, evidence will be required from either the wider HfH program or the literature. Long term collection of a minimum data set for all potential costs and benefits, but specifically for low-probability high-value events would assist the economic evaluation of HfH and similar programs, such as the NSW Aboriginal Communities Water and Sewerage Program (ACWSP) [59].

Quantitative evidence of the program's effect will not be available for all potential benefits. For example, affirmative enquiry undertaken with the tenant workers described personal benefits of “improved self-esteem, new personal relationships, enhanced work ethic, enjoyment out of working and helping others on a worthwhile project” [60], clearly reflecting a potential well-being benefit. Informal evidence also supports the probability of positive well-being benefits arising from tenants' improved sense of self-worth, reduced stress from safety improvements, reduced financial stress from improved energy efficiency, etc. In the absence of a validated instrument to capture these benefits, such outcomes should be acknowledged as intangible benefits of the HfH study project.

The review demonstrated that program costs are more readily and consistently incorporated within economic analyses due to the availability of expenditure data, although issues remain. Implementation costs are neglected in economic analyses of public health interventions, despite their relevance within most programs [61]. The participatory and community-partnered implementation of the HfH program represents a strength that is fully costed, in contrast to many alternative programs. Similarly, another strength of the program is that it does not require on-going support to deliver the intervention. Once the physical infrastructure is upgraded the marginal benefits should be realised. This lies in contrast to behavioural interventions, such as smoking or nutrition, that often require support following an intervention for the assumed benefits to be realised [62,63]. This consideration carries implications for the respective decay rate (dissipation) of their assumed benefits. It is important that the results of the program's economic evaluation reflect the relative sustainability of the benefits from the HfH program, particularly in comparison to alternative programs requiring on-going expenditure.

The economic analyses identified through the review were positioned within an equity policy framework but did not incorporate equity within quantification of any benefit estimates. This is consistent with the immaturity of research methods in this field [64,65] and the absence of definitive and accepted government guidance in this regard [14,19,20,55]. Equity will be addressed through comparison *to* alternative programs or investments seeking to realise comparable objectives. The NSW Treasury supports this approach [12].

This study has limitations. Scoping literature reviews risk selection bias and may not identify all relevant studies, nor do they provide a formal evaluation of evidence quality. Nevertheless, the review is considered a proportionate approach to address the aims and inform the economic protocol. The conduct of the outlined economic evaluation is not funded at time of publication. If policy makers promote the requirement for economic evaluation, it is imperative that independent funding is made available for this task or a percentage of the program funding is quarantined for such activities in the original funding approval.

## 5 Conclusion

Considerable evidence exists regarding the role housing plays in the determination of health and well-being outcomes. The HfH program represents an environmental health initiative funded and administered by NSW Health, that addresses health-related hardware in residential accommodation to improve the potential health outcomes for residents. The purpose of an economic analysis is to provide evidence regarding the relative value of this program, to enable comparison and optimisation of investment into public health.

The results of the review demonstrated that relatively few economic analyses of housing/health programs have been conducted. Of those identified, a wide array of economic methods have been applied, capturing a disparate and often partial mix of costs and benefits. As a result, the economic evidence of the relative value of alternative initiatives lacks clarity and inhibits comparison. Founded on the requirement for decision makers, and the insights from the literature review, the paper outlines a protocol for a cost-benefit analysis that accounts for the disparate health, social, economic and intangible benefits potentially generated from the HfH program. The review and the protocol consequently also establish a more consistent and comprehensive framework for the economic evaluation of comparable housing/health programs going forward.

## Ethics declaration

Review and/or approval by an ethics committee was not needed for this study because the paper reviews economic evaluation methods for interventions designed to improve the health of residents through improvements to housing assets. There were no data collected on human subjects.

## Data availability Statement

No data was collected for the research described in the article.

## Funding sources

This study was funded by a research grant from the Mid-North Coast Local Health District, NSW, Australia. The Housing for Health Project was funded by 10.13039/501100008810Health Protection NSW, NSW, Australia.

## CRediT authorship contribution statement

**Simon Deeming:** Investigation, Methodology, Visualization, Writing – review & editing. **Kerryn Lawrence:** Conceptualization, Funding acquisition, Project administration, Validation, Writing – review & editing. **Jeffrey C. Standen:** Conceptualization, Funding acquisition, Supervision, Validation, Writing – review & editing.

## Declaration of competing interest

The authors declare the following financial interests/personal relationships which may be considered as potential competing interests:

Jeffrey Standen and Kerryn Lawrence are employed by NSW Ministry of Health and manage and implement the Housing for Health program in NSW.

NSW Ministry of Health engaged Simon Deeming Hunter Medical Research Institute to undertake the scoping literature review.

## Appendix A Literature search strategy

Database: Ovid MEDLINE(R) and Epub Ahead of Print, In-Process & Other Non-Indexed Citations and Daily <1946 to September 23, 2019>

Search Strategy.

1 Public housing/or Housing/st
2 ((public or affordable or social or community) adj1 housing). ti,ab,kw,kf.
3 (“low income housing" or “low cost housing"). ti,ab,kw,kf.
4 (health adj2 hous*). ti,ab,kw,kf.
5 ((housing or household*) adj10 (indigenous or aboriginal* or First Nation*)). ti,ab,kw,kf.
6 1 or 2 or 3 or 4 or 5
7 exp Continental Population Groups/
8 (aborigin* or indigenous or torres strait or first nation* people*). ti,ab,kw,kf.
9 7 or 8
10 6 and 9
11 Housing/ec
12 exp “Costs and cost analysis"/
13 (economic* adj1 (evaluat* or analys*)). ti,ab,kw,kf.
14 (cost* adj (effectiv* or utilit* or benefit* or minimi* or analy* or offset* or stud* or consequence or outcome or outcomes)). ti,ab,kw,kf.
15 Program evaluation/
16 (housing adj2 economic*). ti,ab,kw,kf.
17 (economic* adj1 benefit*). ti,ab,kw,kf.
18 11 or 12 or 13 or 14 or 15 or 16 or 17
19 6 and 18
20 Health status/
21 Health status indicators/
22 health status. ti,ab,kw,kf.
23 health impact assessment*.ti,ab,kw,kf.
24 Population health/or Public health/
25 (health adj1 outcome*). ti,ab,kw,kf.
26 20 or 21 or 22 or 23 or 24 or 25
27 6 and 26
28 10 or 19 or 27
29 Developing Countries. sh,kf.
30 (Africa or Asia or Caribbean or “West Indies" or “South America" or “Latin America" or “Central America"). hw,kf,ti,ab,cp.
31 (Afghanistan or Albania or Algeria or Angola or Antigua or Barbuda or Argentina or Armenia or Armenian or Aruba or Azerbaijan or Bahrain or Bangladesh or Barbados or Benin or Byelarus or Byelorussian or Belarus or Belorussian or Belorussia or Belize or Bhutan or Bolivia or Bosnia or Herzegovina or Hercegovina or Botswana or Brasil or Brazil or Bulgaria or Burkina Faso or Burkina Fasso or Upper Volta or Burundi or Urundi or Cambodia or Khmer Republic or Kampuchea or Cameroon or Cameroons or Cameron or Camerons or Cape Verde or Central African Republic or Chad or Chile or China or Colombia or Comoros or Comoro Islands or Comores or Mayotte or Congo or Zaire or Costa Rica or Cote d'Ivoire or Ivory Coast or Croatia or Cuba or Cyprus or Czechoslovakia or Czech Republic or Slovakia or Slovak Republic or Djibouti or French Somaliland or Dominica or Dominican Republic or East Timor or East Timur or Timor Leste or Ecuador or Egypt or United Arab Republic or El Salvador or Eritrea or Estonia or Ethiopia or Fiji or Gabon or Gabonese Republic or Gambia or Gaza or Georgia Republic or Georgian Republic or Ghana or Gold Coast or Greece or Grenada or Guatemala or Guinea or Guam or Guiana or Guyana or Haiti or Honduras or Hungary or India or Maldives or Indonesia or Iran or Iraq or Isle of Man or Jamaica or Jordan or Kazakhstan or Kazakh or Kenya or Kiribati or Korea or Kosovo or Kyrgyzstan or Kirghizia or Kyrgyz Republic or Kirghiz or Kirgizstan or Lao PDR or Laos or Latvia or Lebanon or Lesotho or Basutoland or Liberia or Libya or Lithuania or Macedonia or Madagascar or Malagasy Republic or Malaysia or Malaya or Malay or Sabah or Sarawak or Malawi or Nyasaland or Mali or Malta or Marshall Islands or Mauritania or Mauritius or Agalega Islands or Mexico or Micronesia or Middle East or Moldova or Moldovia or Moldovian or Mongolia or Montenegro or Morocco or Ifni or Mozambique or Myanmar or Myanma or Burma or Namibia or Nepal or Netherlands Antilles or New Caledonia or Nicaragua or Niger or Nigeria or Northern Mariana Islands or Oman or Muscat or Pakistan or Palau or Palestine or Panama or Paraguay or Peru or Philippines or Philipines or Phillipines or Phillippines or Poland or Portugal or Puerto Rico or Romania or Rumania or Roumania or Russia or Russian or Rwanda or Ruanda or Saint Kitts or St Kitts or Nevis or Saint Lucia or St Lucia or Saint Vincent or St Vincent or Grenadines or Samoa or Samoan Islands or Navigator Island or Navigator Islands or Sao Tome or Saudi Arabia or Senegal or Serbia or Montenegro or Seychelles or Sierra Leone or Slovenia or Sri Lanka or Ceylon or Solomon Islands or Somalia or South Africa or Sudan or Suriname or Surinam or Swaziland or Syria or Tajikistan or Tadzhikistan or Tadjikistan or Tadzhik or Tanzania or Thailand or Togo or Togolese Republic or Tonga or Trinidad or Tobago or Tunisia or Turkey or Turkmenistan or Turkmen or Uganda or Ukraine or Uruguay or USSR or Soviet Union or Union of Soviet Socialist Republics or Uzbekistan or Uzbek or Vanuatu or New Hebrides or Venezuela or Vietnam or Viet Nam or West Bank or Yemen or Yugoslavia or Zambia or Zimbabwe or Rhodesia). hw,kf,ti,ab,cp. (3559809)
32 ((developing or less* developed or under developed or underdeveloped or middle income or low* income or underserved or under served or deprived or poor*) adj (countr* or nation? or population? or world)). ti,ab.
33 ((developing or less* developed or under developed or underdeveloped or middle income or low* income) adj (economy or economies)). ti,ab.
34 (low* adj (gdp or gnp or gross domestic or gross national)). ti,ab.
35 (low adj3 middle adj3 countr*). ti,ab.
36 (lmic or lmics or third world or lami countr*). ti,ab.
37 transitional countr*.ti,ab.
38 29 or 30 or 31 or 32 or 33 or 34 or 35 or 36 or 37
39 28 not 38
40 (developed countries or european union). af.
41 europe/or andorra/or austria/or belgium/or exp france/or exp germany/or exp united kingdom/or greece/or ireland/or exp italy/or liechtenstein/or luxembourg/or monaco/or netherlands/or portugal/or exp “scandinavian and nordic countries"/or spain/or switzerland/or exp australia/or new zealand/
42 north america/or exp canada/or exp united states/(1456761)
43 (united kingdom or england or scotland or wales or denmark or finland or iceland or norway or sweden). af.
44 (north america or canada or oecd or united states). af.
45 (europe or andorra or austria or belgium or france or germany or greece or ireland or italy or liechtenstein or luxembourg or monaco or netherlands or portugal or spain or switzerland or australia or new zealand). af.
46 40 or 41 or 42 or 43 or 44 or 45
47 39 and 46
48 limit 47 to (english language and yr = "1996 -Current")
49 limit 48 to (comment or editorial or letter or news)
50 48 not 49
